# P04.08. Predictors of dietary supplement (DS) use among persons receiving home health care: findings from the 2007 national home and hospice care survey (NHHCS)

**DOI:** 10.1186/1472-6882-12-S1-P278

**Published:** 2012-06-12

**Authors:** A Bercovitz, R Nahin, M Sengupta

**Affiliations:** 1 NCHS/CDC, Hyattsville, USA; 2 National Center for Complementary and Alternative Medicine, Bethesda, USA

## Purpose

Each day in the United States in 2007 there were almost 1.5 million home health care patients. This study examines the use of DSs and factors associated with their use in this population.

## Methods

Data were from the 2007 NHHCS - a nationally representative probability sample survey. Medical records of 4,683 current home health patients (Weighted N=1,459,900) were sampled from 677 participating home health agencies (Weighted N=12,300). Therapeutic classes analyzed were Nutritional Products (NP) (vitamins and minerals alone or in combination) and Non-vitamin, Non-mineral Dietary Supplements (NVNMDS). Multivariate analyses were used to examine the independent roles of demographic and health factors with use of NP and NVNMDS, and the association of NP and NVNMDS with pain and pain management strategies. Variables in the multivariate analyses were age, sex, race, number of chronic conditions, whether the patient lived alone and/or had a caregiver, mobility, cognitive impairment, medication assistance and primary payor.

## Results

In preliminary analyses 59% of patients had at least one type of NP and 7% had NVNMDS noted on their medical record. In multivariate analyses, use of NPs was associated with age, sex, payor and number of chronic conditions, and use of NVNMDS was associated with age and race. Neither NP nor NVNMDS were associated with reported pain or a standing order (SO) for pain management. NP, but not NVNMDS, were associated with an “as needed” (PRN) order for pain management.

## Conclusion

Patients who were older, female, with Medicare as primary payor and more chronic conditions were more likely to use NP. Older and white patients were more likely to use NVNMDS. Patients using NP were more likely to have a PRN order, but not a SO. Continuing analyses will include more detailed coding of DSs and analysis of concomitant use of DS with over-the-counter and prescription medications.

